# Supplementary material for: Importance of miRNA stability and alternative primary miRNA isoforms in gene regulation during Drosophila development
Source: eLife. 2018 Jul 19;7:e38389. doi: 10.7554/eLife.38389 (PMC6066331; doi:10.7554/eLife.38389)
Supplement: Transparent reporting form [file elife-38389-transrepform.docx]

***eLife’s* transparent reporting form**

We encourage authors to provide detailed information *within their submission* to facilitate the interpretation and replication of experiments. Authors can upload supporting documentation to indicate the use of appropriate reporting guidelines for health-related research (see [EQUATOR Network](http://www.equator-network.org/%20)), life science research (see the [BioSharing Information Resource](https://biosharing.org/)), or the [ARRIVE guidelines](http://www.plosbiology.org/article/info:doi/10.1371/journal.pbio.1000412) for reporting work involving animal research. Where applicable, authors should refer to any relevant reporting standards documents in this form.

If you have any questions, please consult our Journal Policies and/or contact us: [editorial@elifesciences.org](mailto:editorial@elifesciences.org).

**Sample-size estimation**

- You should state whether an appropriate sample size was computed when the study was being designed
- You should state the statistical method of sample size computation and any required assumptions
- If no explicit power analysis was used, you should describe how you decided what sample (replicate) size (number) to use

Please outline where this information can be found within the submission (e.g., sections or figure legends), or explain why this information doesn’t apply to your submission:

Sample-size estimation was not performed when deciding on the sample size and sequencing depth of RNAseq libraries. Sample-size estimation of transcriptome data is a topic of active discussion and there is no clear standard so far to our best knowledge (Ching et al. 2014, doi: 10.1261/rna.046011.114., Liu et al. 2014 doi:10.1093/bioinformatics/btt688). For small RNA libraries, we made three biological replicates, each of which contains ~10-20 million reads mapped to the genome, mainly considering cost-effectiveness within the budget. The total RNA-seq libraries were previously described and we used all available total RNA-seq datasets published by the modENCODE team (Westholm et al. 2014 doi: 10.1016/j.celrep.2014.10.062, Duff et al. 2015 doi:10.1038/nature14475).

**Replicates**

- You should report how often each experiment was performed
- You should include a definition of biological versus technical replication
- The data obtained should be provided and sufficient information should be provided to indicate the number of independent biological and/or technical replicates
- If you encountered any outliers, you should describe how these were handled
- Criteria for exclusion/inclusion of data should be clearly stated
- High-throughput sequence data should be uploaded before submission, with a private link for reviewers provided (these are available from both GEO and ArrayExpress)

Please outline where this information can be found within the submission (e.g., sections or figure legends), or explain why this information doesn’t apply to your submission:

The small RNA libraries were made in biological triplicate with RNA samples prepared on different days. Two sets of libraries were prepared and sequenced together, while another replicate was done independently from the other two sets. The detailed information regarding the replicates is described in the main text (page 5) and the materials and methods section (pages 19-20).

**Statistical reporting**

- Statistical analysis methods should be described and justified
- Raw data should be presented in figures whenever informative to do so (typically when N per group is less than 10)
- For each experiment, you should identify the statistical tests used, exact values of N, definitions of center, methods of multiple test correction, and dispersion and precision measures (e.g., mean, median, SD, SEM, confidence intervals; and, for the major substantive results, a measure of effect size (e.g., Pearson's r, Cohen's d)
- Report exact p-values wherever possible alongside the summary statistics and 95% confidence intervals. These should be reported for all key questions and not only when the p-value is less than 0.05.

Please outline where this information can be found within the submission (e.g., sections or figure legends), or explain why this information doesn’t apply to your submission:

Statistical tests are reported in Figure legends for Figures 3, 6 and 7. Raw numbers and exact p-values are reported on Figure 3C, Figure 5-S3, Figure 7 D/E, Table S4, S5 and S6.

(For large datasets, or papers with a very large number of statistical tests, you may upload a single table file with tests, Ns, etc., with reference to sections in the manuscript.)

**Group allocation**

- Indicate how samples were allocated into experimental groups (in the case of clinical studies, please specify allocation to treatment method); if randomization was used, please also state if restricted randomization was applied
- Indicate if masking was used during group allocation, data collection and/or data analysis

Please outline where this information can be found within the submission (e.g., sections or figure legends), or explain why this information doesn’t apply to your submission:

For expression analysis reported in Figures 1 and 4, their cutoff values are described in the main text (page 6) and Materials & Methods (page 20).

**Additional data files (“source data”)**

- We encourage you to upload relevant additional data files, such as numerical data that are represented as a graph in a figure, or as a summary table
- Where provided, these should be in the most useful format, and they can be uploaded as “Source data” files linked to a main figure or table
- Include model definition files including the full list of parameters used
- Include code used for data analysis (e.g., R, MatLab)
- Avoid stating that data files are “available upon request”

Please indicate the figures or tables for which source data files have been provided:

For graphs in Figures 1, 3 and 7, raw numerical data are provided in tables S2-S6 in the Excel format.
